# Outcomes of extracorporeal membrane oxygenation and cardiopulmonary bypass in children after drowning-related resuscitation

**DOI:** 10.1177/02676591211041229

**Published:** 2021-09-02

**Authors:** Urda Gottschalk, Maria Köhne, Theresa Holst, Ida Hüners, Maria von Stumm, Götz Müller, Veronika Stark, Victoria van Rüth, Rainer Kozlik-Feldmann, Dominique Singer, Jörg S Sachweh, Daniel Biermann

**Affiliations:** 1 Department of Pediatric Cardiology, University Heart & Vascular Center Hamburg, University Medical Center Hamburg-Eppendorf, Hamburg, Germany; 2 Surgery for Congenital Heart Disease, University Heart & Vascular Center Hamburg, University Medical Center Hamburg-Eppendorf, Hamburg, Germany; 3 Department for Cardiovascular Surgery, University Heart & Vascular Center Hamburg, University Medical Center Hamburg-Eppendorf, Hamburg, Germany; 4 Center for Obstetrics and Pediatrics, Section Neonatology and Pediatric Intensive Care Medicine, University Medical Center Hamburg-Eppendorf, Hamburg, Germany

**Keywords:** drowning, pediatric, cardiac arrest, cardiopulmonary resuscitation, pediatric intensive care, extracorporeal life support, extracorporeal membrane oxygenation, cardiopulmonary bypass

## Abstract

Drowning is one of the leading causes of accidental deaths in children worldwide. However, the use of long-term extracorporeal life support (ECLS) in this setting is not widely established, and rewarming is often achieved by short-term cardiopulmonary bypass (CPB) treatment. Thus, we sought to add our experience with this means of support as a bridge-to-recovery or to-decision. This retrospective single-center study analyzes the outcome of 11 children (median 23 months, minimum–maximum 3 months–6.5 years) who experienced drowning and subsequent cardiopulmonary resuscitation (CPR) between 2005 and 2016 and who were supported by veno-arterial extracorporeal membrane oxygenation (ECMO), CPB, or first CPB then ECMO. All but one incident took place in sweet water. Submersion time ranged between 10 and 50 minutes (median 23 minutes), water temperature between 2°C and 28°C (median 14°C), and body core temperature upon arrival in the emergency department between 20°C and 34°C (median 25°C). Nine patients underwent ongoing CPR from the scene until ECMO or CPB initiation in the operating room. The duration of ECMO or CPB before successful weaning/therapy withdrawal ranged between 2 and 322 hours (median 19 hours). A total of four patients (36%) survived neurologically mildly or not affected after 4 years of follow-up. The data indicate that survival is likely related to a shorter submersion time and lower water temperature. Resuscitation of pediatric patients after drowning has a poor outcome. However, ECMO or CPB might promote recovery in selected cases or serve as a bridge-to-decision tool.

## Introduction

Drowning is one of the leading causes of accidental death in children worldwide.^1–3^ The process of drowning leads to respiratory impairment by water entering the airways and to hypothermia, finally resulting in unconsciousness and cardiac arrest due to hypoxia.^4,5^

For the isolated purpose of rewarming, cardiopulmonary bypass (CPB) is commonly applied after drowning in children,^6,7^ whereas extracorporeal life support (ECLS) treatment alone or combined with CPB is not used as frequently.^7,8^ Most observations and recommendations are based on case reports, mixed series with adults, or on multi-institutional experience. Nevertheless, some centers do not even offer ECLS or CPB for this cohort at all, possibly due to a lack of knowledge or because neither the hardware nor the infrastructure for ECLS implantation is provided. ECLS assists circulatory and respiratory function after cardiopulmonary arrest.^9,10^ In addition, it facilitates rewarming after severe hypothermia. Veno-arterial and veno-venous extracorporeal membrane oxygenation (ECMO) may be used for few days up to several weeks.^10^ According to recent studies, ECLS might be a promising, resuscitative strategy for patients after drowning.^11,12^ Burke et al.^13^ examined retrospective data from the Extracorporeal Life Support Organization (ELSO) registry to determine the outcomes of ECLS and risk factors for death in pediatric drowning victims. Their data suggests that survival is higher in those patients cannulated onto ECLS without prior cardiopulmonary resuscitation (CPR) compared to those cannulated with ECPR (71% vs 23% survival, respectively). Furthermore, they identified ECLS placement during CPR, veno-arterial ECMO mode, renal failure, and CPR on ECLS as risk factors for mortality.

Therefore, more data on pediatric patients with ECMO or CPB after drowning is needed to establish evidence-based treatment protocols and identify reliable predictors of good outcomes.

## Methods

This retrospective single-center study analyzes the outcome of 11 children (age: median 23 months, minimum–maximum 3 months–6.5 years) who experienced drowning and subsequent CPR between 2005 and 2016 and who were supported by ECMO or CPB alone; or combined (first CPB then ECMO). All authors obeyed the Declaration of Helsinki, and the ethics committee of the local medical board (Ethikkommission der Ärztekammer Hamburg) approved the retrospective study without the existing informed consent of each individual included in the study (PV5906).

The water temperatures of the drowning sites were obtained by the Institute of Environment and Hygiene Hamburg, Germany, and were determined for the exact day and time of drowning. Medical data from the drowning site and transport to the emergency room were provided by the local fire department and the responsible emergency medical service (EMS) providers. All drowning sites were situated within a radius of approximately 70 km from the hospital. If there was a distance of 10 km or more, the patients were transported to the emergency department primarily by helicopter. In patients warmer than 32°C, resuscitation efforts are stopped after 60 minutes if no return of spontaneous circulation is achieved. Children with a urinary bladder core temperature (UBCT) lower 32°C are considered for ECMO or CPB if parameters (submersion time and water temperature) for a favorable outcome are present. According to institutional guidelines, patients < 30°C body core temperature (BCT) are directly transferred to the operating room (OR) for interdisciplinary assessment to facilitate timely ECMO or CPB implantation without unnecessary time loss. Patients in need of ECMO or CPB are treated according to the protocol established by Skarda et al.^14^ Briefly summarized, this flowchart-based protocol aims at the timely (<30 minutes) recruitment of personnel and equipment, as well as the rapid provision of blood products. Patients with BCT < 30°C qualify for rewarming by ECMO or CPB. *In our institution, in patients* < 10 kg, cannulation via the right head-neck vessels is considered. Patients between 10 and 35 kg receive central cannulation via median sternotomy, whereas cannulation in patients *>* 35 kg is usually achieved via the femoral vessels. In the case of femoral cannulation, an additional perfusion cannula is placed for distal perfusion of the femoral artery. In recent years, we have preferred ECMO over CPB for rewarming because ECMO allows for a potential long-term treatment if needed. For rewarming, a maximum temperature gradient (blood temperature out (venous) vs blood temperature in (arterial)) of 5°C and a rewarming pace of 1.5–2°C/hour is realized. For neuroprotective reasons rewarming is stopped at 33°C, and the patient transferred to intensive care unit (ICU) if hemodynamically stable. In the case of cardiorespiratory failure, ECMO is used as a bridge-to-decision tool. Subsequently, rewarming to normothermia is performed within 48 hours in ICU, and a comprehensive neurological assessment is carried out. Nomenclature and definitions were used according to the Maastricht Treaty position paper of ELSO.^15^ Statistical analysis was done with SPSS Version 22 (SPSS Inc., Chicago, IL, USA). Due to the limited size of the cohort and inhomogeneous characteristics, the data analysis was exclusively descriptive, reporting median and range or minimum-maximum.

## Results

### Patient characteristics at the scene and early emergency service support

**Figure 1.**
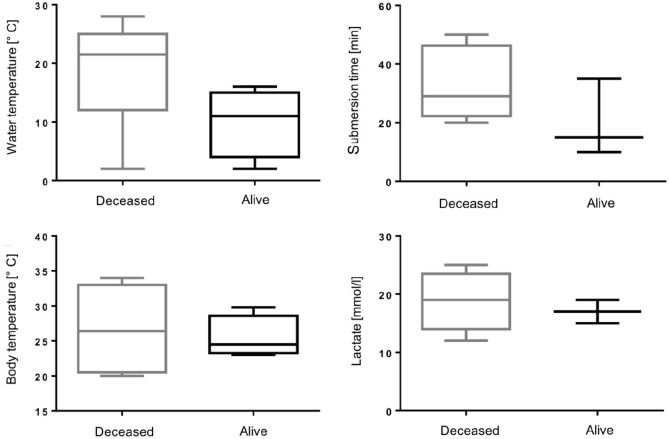
Box plot diagrams of water temperature, submersion time, body temperature and initial lactate value in connection with the drowning incident, deceased (*n* = 7) versus alive (*n* = 4) patients.

Two children fell into freezing lake or pond water during the winter, whereas two others experienced drowning in a warm pool. In the remaining patients, submersion occurred in freshwater during spring and summertime. One patient drowned in saltwater. Water temperature was significantly lower in surviving patients than in deceased patients (survivors median 11°C vs non-survivors median 22°C). Median water temperature was 12°C (range 2°C–28°C). The estimated median submersion duration was 23 minutes (range 10–50 minutes). Submersion time was shorter in children surviving the drowning incident (survivors median 15 minutes vs non-survivors median 29 minutes). All patients showed loss of consciousness as well as circulatory and respiratory arrest with a Glasgow Coma Scale of 3 after salvage from the water. More than half of the patients underwent initial CPR by nonprofessionals (55%) after the rescue. In 45% of the cases, children were discovered and rescued by EMS, allowing for immediate CPR performed by medical professionals. All patients were intubated and manually ventilated at the location of drowning. Initial measurements by EMS yielded a median body temperature of 24°C (range 20°C–34°C) after rescue from the water. In terms of BCT, there were no significant differences found between survivors and non-survivors. Only two of the eleven children regained spontaneous circulation before arrival in the emergency department (ED) or the OR. Patient characteristics related to the drowning incident are listed in Table 1, and essential parameters linked with the event are represented graphically in Figure 1.

**Table 1.** Patient characteristics.

Patients	1	2	3	4	5	6	7	8	9	10	11
Victim information											
Age (month)	3	42	78	77	30	19	22	23	21	20	64
Sex	F	M	M	M	M	F	M	M	F	M	F
Survivor	N	N	N	N	N	N	N	Y	Y	Y	Y
Scene											
Location of drowning	Lake	Pool	Pool	River	Stream	Pond	Lake	Pond	River	River	River
Water temperature (°C)	2	28	25	22	14	12	23	2	16	10	12
Submersion duration (minutes)	20	23	23	35	45	X	50	10	15	X	35
Time to EMS arrival (minutes)	5	8	14	6	19	18	7	22	7	6	10
EMS at location of drowning (minutes)	9	33	50	12	50	60	X	X	20	X	22
First CPR by EMS	Y	N	N	Y	N	N	Y	Y	N	Y	Y
Initial body temperature (°C)	20	33	34	26	21	23	28	23	30	25	24
Initial lactate level (mmol/l)	12	22	25	19	16	X	X	X	15	17	19
ED/OR/ICU											
Time interval to ED (minutes)	4	7	13	10	18	17	6	23	8	5	12
ROSC at arrival ED	Y	N	N	N	N	Y	N	Y	N	N	N
Level of consciousness	Coma	Coma	Coma	Coma	Coma	Coma	Coma	Coma	Coma	Coma	Coma
GCS	3	3	3	3	3	3	3	3	3	3	3
Pupil reflex	Fixed	Fixed	Fixed	Fixed	Fixed	Fixed	Fixed	Fixed	Fixed	Fixed	Fixed
ECLS treatment	ECMO	CPB/ECMO	ECMO	ECMO	CPB/ECMO	CPB/ECMO	CPB	CPB	CPB	CPB/ECMO	CPB
ECLS times (hours)	322	19	20	2	35	34	3	3	2	97	3
Causes of death/severe complications	BD, ARDS, MF	BD, DIC, MF	BD, DIC, MF	MF, DIC	ARDS, MF	BD	MF, DIC	ARDS		ARDS	
Hospital length of stay (days)	14	1	1	1	2	1	1	12	14	32	13

F: female; M: male; N: no; Y: yes; EMS: emergency medical service; CPR: cardiopulmonary resuscitation; ED: emergency department; ROSC: recovery of spontaneous circulation; ECLS: extracorporeal life support; X: missing value; gray: survivors; DIC: disseminated intravascular coagulation; MF: myocardial Failure (after weaning from ECLS); BD: brain death; ARDS: acute respiratory distress syndrome; OR: operating room; ICU: intensive care unit.

### Management after arrival in the emergency department (patients > 30°C)

Initial arterial blood gas analysis performed immediately upon arrival revealed a median lactate level of 18 mmol/l (range 12–25 mmol/l). There were no significant differences between survivors and non-survivors regarding the peak lactate level. The decision to use ECMO or CPB was made on an individual basis for each child (see methods section). In the case of circulatory arrest, hemodynamic instability, or UBCT below 32°C, the use of ECMO or CPB was considered. After an initial interdisciplinary assessment in the emergency department (ED), patients were transferred to the OR. Patients who were reported by EMS with a CBT lower than 30°C were transferred directly to the OR.

### Management in the operating room and the intensive care unit

Arterial blood gas before ECMO or CPB implantation with a median pH (*T_body_*) of 6.8 (range 6.6–6.9), a median lactate level of 18 mmol/l (range 15–48 mmol/l), and a median potassium level of 5 mmol/l (range 4–9 mmol/l) indicated severe shock in all patients. Nine of the children remained in circulatory arrest until the connection to ECMO or CPB. Three patients were connected to venoarterial ECMO, and eight received CPB. Of these eight, four were connected to venoarterial ECMO after failed weaning from CPB due to respiratory or cardiac failure. In most cases (nine patients), surgical access for CPB or ECMO implantation was performed through a median sternotomy using the right atrium for venous and the ascending aorta for arterial cannulation. In one patient, femoral cannulation using the Seldinger’s technique was chosen for ECMO application. In another patient, CPB connection was performed by puncturing the internal jugular vein and the right carotid artery. The overall median duration of ECMO support was 19 hours (range 2–322 hours). Median CPB time was 160 minutes (range 90–194 minutes). During ECMO or CPB rewarming, six (55%) of the eleven patients developed spontaneous circulation after initial cardiac arrest. One patient, who had been put on ECMO, ceased in the OR due to insufficient circulation despite having received the maximum amount of supportive treatment. While being in the ICU, four other patients suffered from diffuse bleeding due to disseminated intravascular coagulation, and four patients developed acute respiratory distress syndrome (ARDS). Other complications were related to cerebral edema, progressive pleural effusion, ventricular failure, and systemic inflammation.

In the current study, four (36%, two males, two females) of the eleven children survived with an almost normal neurological outcome after 4 years of follow-up (all visiting regular schools). Three of them received CPB without ECMO therapy. One child was connected to ECMO after the unsuccessful weaning from CPB. Two of the survivors showed signs of ARDS, which resolved over time in ICU. The remaining seven patients (64%, five males, two females) died from unmanageable complications during or following the CPB or ECMO connection. Heart failure or severe cerebral damage depicted on CT imaging were direct causes of death or led indirectly to the final decision to disconnect from ECMO. The final agreement to pronounce a patient as brain dead was confirmed by a multidisciplinary team led by a neurologist in each case.

## Discussion

Drowning in children is a common incident with a lethal outcome in many cases. There is still no uniform treatment concept for pediatric drowning patients with prolonged resuscitation arriving in the ED. Due to improved survival rates and fewer complications over the last two decades, ECMO application is nowadays increasingly recommended as resuscitative therapy for cardiac failure or arrest.^9,16,17^ According to ELSO, the overall survival in 565 pediatric patients placed on ECMO during resuscitation was 40%.^17^ Advantages of ECMO for children after resuscitation, for example, controlled rewarming and cardiopulmonary support, were only reported in case reports examining a small number of patients on an individual basis.^4,9,18,19^ For the treatment of hypothermia, the International Liaison Committee on Resuscitation^20^ and the American Heart Association^21^ currently recommend specialized therapy even if the child remains in a comatose state after initial resuscitation. Primary hypothermia might have an influence on survival for neuroprotective reasons, yet, according to some reports, low water temperature is not always associated with higher survival rates.^16,22^

In our study, we found that children who drowned in cooler water were more likely to survive than those who drowned in warmer water. The four children who survived the incident drowned in water whose temperature was below 16°C. We assume that irrespective of the achieved BCT, which was found to be similar in survivors and non-survivors, brain temperature is more rapidly lowered under these conditions, and the odds for survival are thus increased. Furthermore, a longer submersion time seemed to be related to an unfavorable outcome. Still, the longest submersion time in the group of survivors was 35 minutes. This is especially surprising as commonly suggested cut-off submersion times for death or severe irreversible cerebral damage due to hypoxia range between 10 and 20 minutes only.^4,22,23^ The initial blood-lactate values immediately after the rescue, were not clearly correlated with survival or death in our study.

The most common cause of death in hospitalized drowning patients is severe hypoxic cerebral damage.^24^ In our cohort, ECMO or CPB was stopped either in patients with severe hypoxic cerebral damage (four cases) or in patients with persisting myocardial failure (three cases). With respect to the high potential of dismal neurological outcome after drowning and prolonged resuscitation, it is remarkable that all survivors of our cohort participate in regular school without gross signs of neurological pathology. This may be related to the high number of patients in our cohort being resuscitated by professionals after salvage from the water and the short responding times of the local fire department. However, specialized neurological assessment of the patients was not conducted, and all had neuropediatric rehabilitation after discharge from the hospital.

Cardiopulmonary bypass, and ECMO in particular, enables slow and controlled rewarming, possibly having a supportive effect on cerebral and metabolic regulation even in the case of hypoxic brain damage.^11^ However, in our cohort, all survivors were treated with CBP alone with only one patient with ECMO implantation after CPB treatment in the ICU. The survival of drowned patients is dependent upon time management and the efficiency of resuscitation. Thus, the time for assessment of the patient’s status before treatment should be kept as short as possible in order to minimize the period until initiation of ECMO or CPB. Initial evaluation of drowned patients in the OR instead of the ED potentially avoids time-consuming transportation from one location to the other. As outlined in the methods section, our institution developed clear instructions for action if children under resuscitation are announced by EMS.

The evaluation of standardized protocols for the therapy of pediatric patients who experienced drowning still encounters many challenges, especially within the context of ECMO treatment. Our study is limited by its retrospective nature and the limited patient sample size. Consequently, multi-center studies are needed to allow for data comparability and more accurate statistical results.

In conclusion, resuscitation of pediatric patients after drowning has a poor outcome. However, ECMO or CPB might promote cardiopulmonary recovery in selected cases after cold water drowning or serve as a valuable life-saving bridge-to-decision tool.
